# Enhancement of Quercetin-Induced Apoptosis by Cotreatment with Autophagy Inhibitor Is Associated with Augmentation of BAK-Dependent Mitochondrial Pathway in Jurkat T Cells

**DOI:** 10.1155/2019/7989276

**Published:** 2019-11-15

**Authors:** Eun Ji Ha, Ki Yun Kim, Chae Eun Kim, Do Youn Jun, Young Ho Kim

**Affiliations:** ^1^Laboratory of Immunobiology, School of Life Science and Biotechnology, College of Natural Sciences, Kyungpook National University, Daegu, Republic of Korea; ^2^Astrogen Inc., Techno-Building 313, Kyungpook National University, Daegu 41566, Republic of Korea

## Abstract

A flavonoid antioxidant quercetin promotes dose-dependent activation of the ATM-CHK-p53 pathway, downregulation of antiapoptotic survivin, and upregulation of proapoptotic NOXA in human T cell acute lymphoblastic leukemia Jurkat clones (J/Neo and J/BCL-XL). However, the downregulation of antiapoptotic BAG3 and MCL-1 occurred in J/Neo cells but not in J/BCL-XL cells overexpressing BCL-XL. Additionally, several BCL-XL-sensitive intrinsic mitochondrial apoptotic events including apoptotic sub-G_1_ cell accumulation, TUNEL-positive DNA fragmentation, BAK activation, mitochondrial membrane potential (*Δψ*m) loss, caspase-9/caspase-8/caspase-3 activation, and PARP cleavage were induced only in J/Neo cells. Both cytosolic and mitochondrial ROS levels were elevated in quercetin-treated J/Neo cells; however, the ROS elevations were almost completely abrogated in J/BCL-XL cells, suggesting the ROS elevations were downstream of BCL-XL-sensitive mitochondrial damage and dysfunction. Wild-type A3, FADD-deficient I2.1, and caspase-8-deficient I9.2 Jurkat clones exhibited similar susceptibilities to the cytotoxicity of quercetin, excluding an involvement of extrinsic pathway in triggering the apoptosis. The autophagic events such as attenuation of AKT-mTOR pathway, formation of acridine orange-stainable acidic vesicular organelles, conversion of microtubule-associated protein 1 light chain 3-I (LC3-I) to LC3-II, and downregulation of p62/SQSTM1 level were detected in quercetin-treated J/Neo and J/BCL-XL cells, regardless of BCL-XL overexpression. Cotreatment with the autophagy inhibitor (3-methyladenine, LY294002, or chloroquine) resulted in a significant enhancement of quercetin-induced BAK activation and subsequently the mitochondrial damage-mediated apoptosis pathway by augmenting the downregulation of BAG3 and MCL-1 levels in J/Neo cells. These results demonstrated that quercetin induces intrinsic apoptosis and cytoprotective autophagy, and autophagy inhibition can potentiate BAK-dependent apoptotic activity of quercetin in Jurkat T cells.

## 1. Introduction

Human T cell acute lymphoblastic leukemia (T-ALL), originating from the malignant transformation of T cell lineage lymphoblasts, is an aggressive neoplasm and is responsible for approximately 20% of all ALL cases [1, 2]. Recent advances in risk-adapted chemotherapy regimens have improved overall survival rates in both childhood and adult T-ALL. However, resistance to chemotherapy and early relapse can occur in T-ALL with high-risk features, leading to unfavorable prognosis and low survival rates [3]. To improve overall survival rates in chemotherapeutic treatment of T-ALL, novel antitumor agents that can minimize drug resistance and side effects are urgently required.

The effectiveness of chemotherapy in tumor regression depends largely on the cytostatic and/or cytotoxic effects of chemotherapeutic drugs on tumor cells. Several biochemical mechanisms, such as apoptosis, necrosis, and autophagy, have been implicated in chemotherapy-mediated cytotoxicity toward tumor cells [4–6]. Among these, apoptosis is the most efficient mechanism by which malignant tumor cells can be eliminated following chemotherapy treatment [7, 8]. In chemotherapy-induced apoptosis of tumor cells, two distinctive death signaling pathways are involved: the intrinsic mitochondria-dependent pathway [9] and the extrinsic death receptor-dependent pathway [10]. The former pathway is mainly associated with tumor cell apoptosis provoked by chemotherapy drugs such as DNA- and microtubule-damaging agents [11].

Autophagy is a cellular catabolic degradation process which is responsible for sequestering unnecessary or dysfunctional cellular components via the formation of double-membrane vesicles (autophagosomes) and targeting them for degradation via their fusion with lysosomes to generate single-membrane autolysosomes. Several studies have reported that the autophagic capacity is elevated in tumor cells compared to normal cells [12]. This can promote tumor cell survival under stress conditions such as nutritional deprivation and chemotherapy. Because tumor cells can activate autophagy following cellular stress associated with chemotherapy, the pharmacological inhibition of autophagy may be an effective approach to accelerate tumor cell death induced by chemotherapy drugs [13]. However, the role of autophagy activation, which occurs following treatment with chemotherapy drugs, in regulating cancer cell death or survival remains controversial.

Previously, we sought to isolate a pharmacologically safe apoptogenic substance from a collection of edible plants, as edible plant-derived cytotoxic components to tumors may be less toxic to normal cells [14, 15]. The purification of antitumor ingredients of the grains of *Sorghum bicolor* (L.) *Moench* var. *Hwanggeumchal*, by a serial solvent extraction and silica gel column chromatography, has led to the identification of a flavonoid compound quercetin, which is well-known for its antioxidant and antitumor properties [16–18]. The antitumor effect of quercetin appears to be exerted by cell cycle arrest and apoptotic cell death, which can be mediated by several different mechanisms, including mitochondrial damage- and endoplasmic reticulum (ER) stress-mediated apoptotic pathways. In relation to these quercetin caused activation of apoptotic pathways, topoisomerase II inhibition-mediated DNA damage [19, 20], upregulation of death receptors [21], reactive oxygen species (ROS) generation [21, 22], alteration of intracellular Ca^2+^ via inhibition of sarcoplasmic/endoplasmic reticulum Ca^2+^ ATPase [23–25], and inhibition of signal transducer and activator of transcription 3 (STAT3) signaling pathway have been implicated [26, 27]. Additionally, quercetin could induce apoptosis and protective autophagy in human malignant glioblastoma U373MG cells [28], human aggressive B cell lymphoma [27], and human bladder cancer cells [29]. However, the individual cellular signaling pathways involved in quercetin-induced apoptosis and autophagy and their interrelationship remain obscure. Moreover, little is known about the involvement of the extrinsic apoptotic pathway in quercetin-induced apoptosis or whether the antitumor activity of quercetin might be applicable to T-ALL.

In this study, we examined whether quercetin, which is one of the most abundant phenolic compounds in *S. bicolor* grains, could provoke the DNA damage-caused mitochondrial apoptosis pathway and the cytoprotective autophagy pathway simultaneously and sought to identify regulators of crosstalk between these two pathways in quercetin-treated human T-ALL Jurkat cells. Additionally, to examine the involvement of the extrinsic pathway in quercetin-induced mitochondrial apoptosis, we compared apoptotic sub-G_1_ cell accumulation and *Δψ*m loss among quercetin-treated wild-type (A3), Fas-associated death domain- (FADD-) deficient (I2.1), and caspase-8-deficient (I9.2) Jurkat clones.

## 2. Materials and Methods

### 2.1. Reagents, Chemicals, Cells, and Culture Media

Ethanol (99.9%) and methanol were obtained from Duksan (Seoul, Korea). Quercetin, 3-methyladenine (3-MA), LY294002 (LY), chloroquine (CQ), dimethyl sulfoxide (DMSO), propidium iodide (PI), 3,3′-dihexyloxacarbocyanine iodide (DiOC6), 4′,6-diamidino-2-phenylindole dihydrochloride (DAPI), 3-(4,5-dimethylthiazol-2-yl)-2,5-diphenyl-tetrazolium bromide (MTT), and aminopropyltriethoxy-silane were purchased from Sigma-Aldrich (St. Louis, MO, USA). Dihydroethidium (DHE), a probe specific for detecting reactive oxygen species (ROS), was purchased from Santa Cruz Biotechnology (Santa Cruz, CA, USA). CellROX Deep Red reagent and MitoSOX Red reagent were purchased from Molecular Probes (Carlsbad, CA, USA). The anti-PARP, anti-BAK, anti-BCL-XL, anti-BCL-2, anti-p53, anti-BIM, anti-MCL-1, and anti-p62 antibodies were purchased from Santa Cruz Biotechnology. The anti-p-ATM (Ser-1981), anti-ATM, anti-p-CHK1 (Ser-317), anti-CHK1, anti-p-CHK2 (Ser-19), anti-CHK2, anti-p-p53 (Ser-15), anti-p-PDK1 (Ser-241), anti-PDK1, anti-p-AKT (Thr-308), anti-AKT, anti-p-mTOR (Ser-2448), anti-mTOR, anti-p-ULK (Ser-757), anti-LC3, anti-caspase-8, anti-caspase-9, ant-caspase-3, and anti-BID antibodies were obtained from Cell Signaling Technology (Beverly, MA, USA). The anti-BAG3 antibody was purchased from Abcam (Cambridge, UK), and the anti-GAPDH antibody and acridine orange were purchased from Thermo Fisher Scientific (Waltham, USA). The active conformation-specific anti-BAK (Ab-1) antibody was purchased from Calbiochem (San Diego, CA, USA). Human acute leukemia Jurkat T cell clones which were stably transfected with the vector (J/Neo) or the antiapoptotic *BCL-XL* gene (J/BCL-XL) were provided by Dr. Dennis Taub (Gerontology Research Center, NIA/NIH, Baltimore, MD, USA). Jurkat T cell clones A3, I2.1, and I9.2 were purchased from the American Type Culture Collection (Manassas, VA, USA) and maintained in RPMI 1640 complete medium containing 10% FBS, 20 mM HEPES (pH 7.0), 50 *μ*M *β*-mercaptoethanol, and 100 *μ*g/ml gentamicin. For cultivation of both J/Neo and J/BCL-XL cells, 400 *μ*g/ml G418 was added to RPMI 1640 complete medium.

### 2.2. Extraction and HPLC Analysis of Phenolic Compounds from the Grains of *Sorghum bicolor* (L.) *Moench* var. *Hwanggeumchal*

Ethanol extraction from *Sorghum bicolor* grains was performed as previously described [30], and the dry weights of the 80% ethanol extract and organic solvent fractions are described in Supplementary [Supplementary-material supplementary-material-1].

The contents of phenolic compounds in the 80% ethanol extract of *S. bicolor* grains were analyzed by HPLC (Agilent 1200; Agilent Technologies, Waldbronn, Germany) as described elsewhere [31]. Briefly, the analytical column a ZORBAX ODS analytical column (4.6 × 250 mm; Agilent Technologies) was used with a guard column (Phenomenex, Torrance, CA, USA). The detection wavelength was set at 280 nm, and the solvent flow rate was held constant at 1.0 ml/min. The mobile phase used for the separation consisted of solvent A (0.1% acetic acid in distilled water) and solvent B (0.1% acetic acid in acetonitrile). A gradient elution procedure was used as 0 min 92% A, 2-27 min 90% A, 27-50 min 70% A, 50-51 min 10% A, 51-60 min 0% A, and 60-62 min 92% A. The injection volume used for analysis was 20 *μ*l.

The standard phenolic compounds used for HPLC analysis were biochanin A, caffeic acid, *p*-coumaric acid, *t*-cinnamic acid, gentisic acid, hesperidin, hesperitin, 4-hydroxybenzoic acid, kaempferol, myricetin, naringenin, naringin, protocatechuic acid, quercetin, resveratrol, salicylic acid, and veratric acid (Sigma-Aldrich). The column temperature was set at 60°C, and the effluent was monitored at 279 nm. All samples were analyzed in triplicates.

Among the 17 phenolic compounds detected in the 80% ethanol extract, quercetin, kaempferol, naringenin, gentisic acid, salicylic acid, and resveratrol were the most abundant compounds, and their contents were 9.29, 2.90, 2.60, 2.13, 1.76, and 1.40 *μ*g/mg of the 80% ethanol extract, respectively (Supplementary [Supplementary-material supplementary-material-1]). When the cytotoxic activities of these phenolic compounds were investigated by measuring the IC_50_ values against human T-ALL Jurkat cell lines (J/Neo and A3), quercetin exhibited the strongest cytotoxicity, followed by kaempferol and resveratrol; however, gentisic acid and naringenin exhibited the lowest cytotoxicity (Supplementary [Supplementary-material supplementary-material-1]).

### 2.3. Cytotoxicity Assay

The cytotoxic effect of individual organic solvent extracts of *Sorghum bicolor* grains and six major phenolic compounds (quercetin, kaempferol, naringenin, gentisic acid, salicylic acid, and resveratrol) on Jurkat T cells was assessed by the MTT assay as previously described [8]. Briefly, cells (5.0 × 10^4^/well) were added to a serial dilution of individual samples in 96-well plates (Corning, New York, USA). Following incubation for indicated time periods, MTT solution was added to each well and then incubated for an additional 4 h. The colored formazan crystal generated from MTT was dissolved in DMSO to measure the optical density at 540 nm by a plate reader.

### 2.4. Flow Cytometric Analysis

Flow cytometric analyses of apoptotic alterations in the cell cycle status of cells treated with quercetin were performed as previously described [8]. Detection of apoptotic and necrotic cells was performed using an Annexin V-FITC apoptosis kit (Clontech, Takara Bio Inc., Shiga, Japan) as previously described [8]. Quercetin-induced changes in mitochondrial membrane potential (*Δψ*m) were measured after DiOC_6_ staining [32, 33]. Activation of BAK was detected by the active conformation-specific anti-BAK antibody (Ab-1) [34]. The formation of intracellular acidic vesicular organelles (AVOs) or autolysosome vacuoles was analyzed by flow cytometry with acridine orange staining as previously described [35]. Briefly, cells were incubated with the RPMI 1640 complete medium containing 2 *μ*M acridine orange in a 5% CO_2_ incubator at 37°C for 30 min. Intracellular, cytosolic, and mitochondrial reactive oxygen species (ROS) levels were detected after treatment of the cells with DHE (1 *μ*M), CellROX Deep Red reagent (5 *μ*M), or MitoSOX Red reagent (0.5 *μ*M) in a CO_2_ incubator at 37°C for 30 min, and the fluorescence intensity was analyzed with an Attune NxT Flow Cytometer (Thermo Fisher Scientific, Waltham, MA, USA) as described elsewhere [36].

### 2.5. TdT-Mediated dUTP Nick-End Labeling (TUNEL) Assay

Vehicle (0.1% DMSO)-treated control and quercetin-treated cells were adhered onto glass cover slips pretreated with 2% aminopropyltriethoxy-silane as previously described [34]. The cells were then subjected to fluorescence-TUNEL assay using the In Situ Cell Death Detection Kit (Roche Applied Science, Basel, Switzerland) [36]. To observe the nuclei, the cells were stained with DAPI and examined under the LSM 700 confocal laser scanning microscope (Carl Zeiss MicroImaging GmbH, Germany).

### 2.6. Preparation of Cell Lysates and Western Blot Analysis

Following cell lysate preparation, an equivalent amount of protein lysate (20–25 *μ*g) was electrophoresed on a 4–12% NuPAGE gradient or 7% NuPAGE gels with MOPS buffer or Tris-Acetate Buffer and then electrotransferred to a nylon membrane as described elsewhere [8]. Protein detection was performed using an ECL western blot kit (Amersham, Arlington Heights, IL, USA). Densitometry was performed using ImageQuant TL software (Amersham) as previously described [34]. The arbitrary densitometric units for each protein of interest were normalized to the densitometric units for GAPDH.

### 2.7. Statistical Analysis

Each result is a representative of at least three separate experiments, unless otherwise indicated. Statistical analysis was processed using Student's *t*-test to evaluate the significance of differences between two groups and one-way ANOVA between three or more groups as previously described [36]. *P* values < 0.05 were considered significant. Statistical analysis was conducted using the SPSS Statistics version 23 (IBM, Armonk, NY, USA).

## 3. Results and Discussion

### 3.1. Cytotoxicity of Quercetin in J/Neo and J/BCL-XL Cells

**Figure 1:**
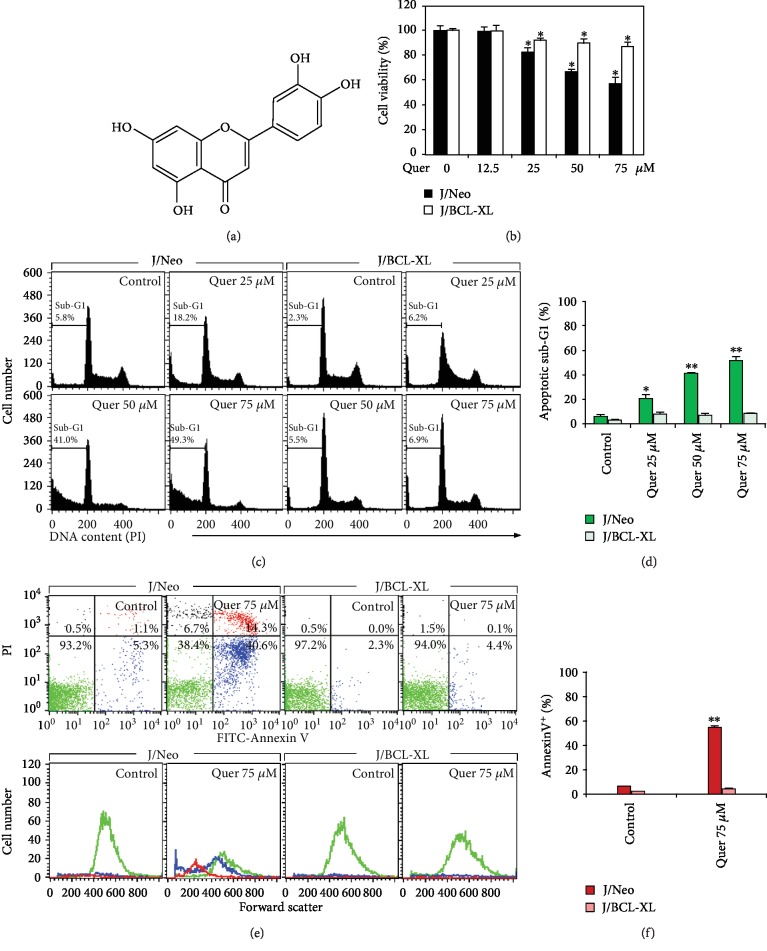
Cytotoxicity of quercetin toward Jurkat T cells was mainly exerted by the induction of BCL-XL-sensitive apoptosis without necrosis. (a) The chemical structure of quercetin. (b) For cell viability analysis, individual cells (5 × 10^4^/well) were incubated with vehicle (0.1% DMSO) or quercetin at indicated doses in a 96-well plate for 11 h and further incubated with MTT for 4 h to measure cell viability. Data are expressed as means ± SD (*n* = 3 with three replicates per independent experiment). (c, d) Cell cycle distribution was measured by flow cytometric analysis with PI staining. (e, f) Annexin V-positive apoptotic cells were determined by flow cytometric analysis with FITC-Annexin V/PI double staining. The forward scatter properties of unstained live, early apoptotic, and late apoptotic cells were measured to analyze alterations in cell size during the induced apoptosis. A representative study is shown and two additional experiments yielded similar results. All data in bar graphs represent the means of triplicate experiments. Error bars represent standard deviations with ^∗^ and ^∗∗^ indicating *P* < 0.05 and *P* < 0.01, respectively, compared with the control.

To examine whether the intrinsic mitochondria-dependent apoptosis induction, which can be prevented by BCL-XL overexpression, is crucial for the cytotoxicity of quercetin (Figure 1(a)), the cytotoxic effects of quercetin on J/Neo and J/BCL-XL cells were compared. As measured by the MTT assay, the viabilities of J/Neo cells in the presence of 12.5, 25, 50, and 75 *μ*M quercetin were 99.5%, 82.5%, 66.9%, and 57.3%, whereas those of J/BCL-XL cells were 99.6%, 91.7%, 89.8%, and 88.1%, respectively (Figure 1(b)). The effect of quercetin on cell cycle distribution of J/Neo and J/BCL-XL cells was compared by flow cytometric analysis. As shown in Figures 1(c) and 1(d), the percentage of sub-G_1_ cells, which represent apoptotic cells undergoing internucleosomal DNA fragmentation [37], was enhanced in quercetin-treated J/Neo cells in a dose-dependent manner; however, the enhancement was not detected in J/BCL-XL cells. These results demonstrated that the cytotoxicity of quercetin was mainly attributed to apoptosis induction which could be completely blocked by BCL-XL overexpression.

During apoptosis induction, cells undergo various morphological changes, including cellular shrinkage and external exposure of phosphatidylserine on the cytoplasmic membrane, whereas necrosis is accompanied by cellular swelling and dilation of organelles, resulting in the plasma membrane ruptures [38]. Previously, it has also been shown that necrotic cells, early apoptotic cells, and late apoptotic cells are different in their FITC-Annexin V/PI dual staining patterns [39]. In these contexts, to elucidate whether quercetin-induced enhancement of the apoptotic sub-G_1_ cell percentage in J/Neo cells was caused by apoptosis or apoptosis accompanying necrosis, the cells were analyzed by flow cytometry using FITC-Annexin V and PI staining.

**Figure 2:**
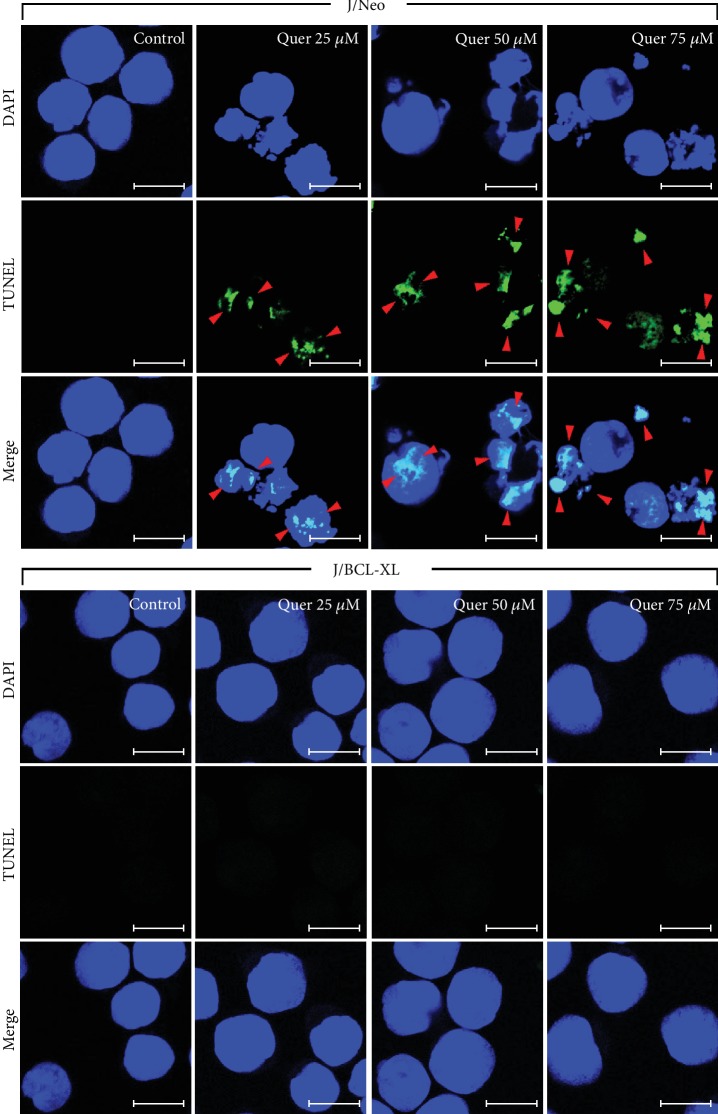
TUNEL-positive DNA fragmentation was observed in quercetin-treated J/Neo cells in a dose-dependent manner but not in J/BCL-XL cells. Individual cells were incubated at a density of 5 × 10^5^/ml with vehicle or indicated concentrations of quercetin for 11 h. Apoptotic DNA fragmentation analysis was performed using a TUNEL assay as described in Materials and Methods. Symbols: red arrowhead: TUNEL-positive DNA fragmentation. The scale bar represents a length of 10 *μ*m in the images. A representative study is shown; two additional experiments yielded similar results.

When J/Neo cells were treated with 75 *μ*M quercetin for 11 h, the level of necrotic cells (stained with only PI) was negligible (Figures 1(e) and 1(f)). Simultaneously, the early apoptotic cells (stained with only FITC-Annexin V) as well as late apoptotic cells (stained with both FITC-Annexin V and PI) were elevated to the levels of 40.6% and 14.3%, respectively. Although the unstained live cells following quercetin treatment exhibited no change in their light scattering properties, early and late apoptotic cells showed commonly a decrease in forward scatter, demonstrating typical apoptotic cellular shrinkage rather than necrotic cellular swelling. In contrast, there was no increase in the rate of either early apoptotic or late apoptotic cells in quercetin-treated J/BCL-XL cells. Whereas J/Neo cells following treatment with 25, 50, and 75 *μ*M quercetin clearly showed TUNEL-positive nuclei dose-dependently compared with control cells, quercetin-treated J/BCL-XL cells failed to show TUNEL-positive cells (Figure 2).

Consequently, these results demonstrated that exposure of Jurkat T cells to quercetin (25–75 *μ*M) caused apoptotic cell death in a dose-dependent manner and that the cytotoxicity of quercetin toward Jurkat T cells could be attributed mainly to BCL-XL-sensitive apoptotic DNA fragmentation, but not to necrosis.

### 3.2. Induction of the Intrinsic Mitochondrial Apoptosis by Quercetin

**Figure 3:**
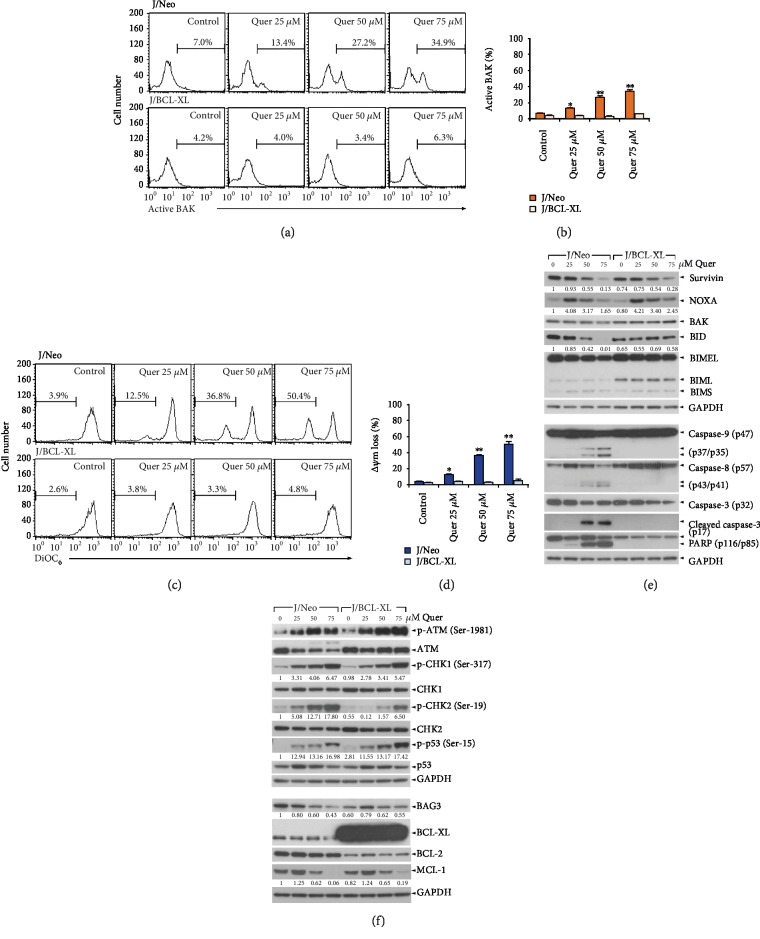
Quercetin induces mitochondria-dependent apoptotic events and DNA damage-mediated activation of the ATM-CHK-p53 pathway in Jurkat T cells. (a, b) BAK activation and (c, d) *Δψ*m loss were determined by flow cytometry in J/Neo and J/BCL-XL cells treated with vehicle or quercetin at indicated doses for 11 h. (e, f) Total cell lysates from equivalent cultures were prepared, and western blot analyses of survivin, NOXA, BAK, BID, BIM, caspase-9, caspase-8, caspase-3, PARP, p-ATM (Ser-1981), ATM, p-CHK1 (Ser-317), CHK1, p-CHK2 (Ser-19), CHK2, p-p53 (Ser-15), p53, BAG3, BCL-XL, BCL-2, MCL-1, and GAPDH were carried out as described in Materials and Methods. A representative study is shown and two additional experiments yielded similar results. Error bars represent standard deviations with ^∗^ and ^∗∗^ indicating *P* < 0.05 and *P* < 0.01, respectively, compared with the control.

Mitochondrial cytochrome *c* release into the cytosol and subsequent activation of the caspase cascade are frequently associated with chemotherapy-induced apoptosis in tumor cells [40]. Additionally, antiapoptotic proteins (BCL-2, BCL-XL, and MCL-1) are known to protect cells from apoptosis by blocking the activation of proapoptotic BCL-2 family proteins BAK and BAX, which causes permeabilization of the mitochondrial outer membrane, *Δψ*m loss, and cytochrome *c* efflux [41]. To examine the contribution of BAK/BAX activation and *Δψ*m loss to the quercetin-induced apoptosis, we carried out flow cytometric analyses of quercetin-treated cells using active conformation-specific anti-BAK (Ab-1) and anti-BAX (6A7) antibodies for detection of active BAK and BAX and using DiOC_6_ staining for detection of *Δψ*m loss. After J/Neo cells were treated with quercetin (25, 50, and 75 *μ*M) for 11 h, the levels of J/Neo cells showing BAK activation appeared to be 13.4%, 27.2%, and 34.9%, respectively, whereas the BAK activation was negligible in J/BCL-XL cells (Figures 3(a) and 3(b)). By contrast, the activation of BAX was not detected in either J/Neo or J/BCL-XL cells after quercetin treatment (data not shown). Under these conditions, quercetin-treated J/Neo cells exhibited *Δψ*m loss in a dose-dependent manner; however, the *Δψ*m loss was completely abrogated in J/BCL-XL cells (Figures 3(c) and 3(d)). These results demonstrated that quercetin-induced *Δψ*m loss was promoted by the BAK activation, which could be blocked by BCL-XL overexpression.

The activation of BAK and resultant *Δψ*m loss were reported to be upstream events of the mitochondrial cytochrome *c* release into the cytosol, mediating the activation of multiple caspases such as caspase-9 and caspase-3 [42]. In this regard, it was highly likely that the activation of caspase-9 and caspase-3, which is a prerequisite for quercetin-induced apoptosis, fails to occur in J/BCL-XL cells due to antiapoptotic action of overexpressed BCL-XL. To test this prediction, we performed western blot analyses to determine whether caspase cascade activation occurred in J/Neo cells, but not in J/BCL-XL cells, after quercetin treatment. As shown in Figure 3(e), caspase-9 activation through proteolytic degradation of an inactive procaspase-9 (47 kDa) into its active forms (37/35 kDa) and caspase-3 activation through proteolytic degradation of a 32 kDa proenzyme into its 17 kDa active form were detected in quercetin-treated J/Neo cells dose-dependently. The activation of caspase-8 through proteolytic cleavage of proenzyme (57 kDa) into active forms (43/41 kDa) was also enhanced in J/Neo cells following quercetin treatment. Simultaneously, the level of BID protein (22 kDa), which is known to be degraded by active caspase-8 to generate the truncated BID (tBID, 15 kDa) causing mitochondrial cytochrome *c* release into the cytosol [43, 44], appeared to decrease in quercetin-treated J/Neo cells. However, the generation of tBID was not observed by western blot analysis in quercetin-treated J/Neo cells, presumably due to the short half-life of tBID. In agreement with caspase-3 activation, PARP cleavage was observed in J/Neo cells. However, all of these apoptotic responses caused by quercetin treatment were completely abrogated in J/BCL-XL cells.

In relation to antitumor activity of quercetin, inhibition of topoisomerase II and subsequent induction of DNA damage-caused apoptosis were implicated [19, 20]. Several studies have reported that apoptosis provoked by DNA damage like DNA double-strand break is exerted via induction of the ATM/ATR-CHK1/2-p53 pathway, leading to alterations of proapoptotic and antiapoptotic protein levels [45, 46]. Consistent with these studies, the elevation of phosphorylation state of ATM at Ser-1981, CHK1 at Ser-317, CHK2 at Ser-19, and p53 at Ser-15, which represents their activation possibly resulting from the quercetin-mediated topoisomerase II inhibition and resultant DNA damage, was observed in both J/Neo and J/BCL-XL cells treated with quercetin (25–75 *μ*M) in a dose-dependent manner (Figure 3(f)). The downstream events of the ATM/ATR-CHK1/2-p53 pathway, upregulation of proapoptotic NOXA level, and downregulation of antiapoptotic survivin level occurred in quercetin-treated J/Neo and J/BCL-XL cells, whereas antiapoptotic BAG3 and MCL-1 levels appeared to decrease only in J/Neo cells. Under these conditions, the BCL-2 and BCL-XL levels were not markedly altered in both cell types following quercetin treatment, as were neither the electrophoretic mobility reduction of BIM during SDS-polyacrylamide gel electrophoresis, exhibiting its activation [34], nor an increase of BAK level. Previously, it was reported that quercetin-mediated downregulation of MCL-1 and survivin rendered non-Hodgkin's lymphoma B cells susceptible to TRAIL-induced apoptosis [47]. These previous and current results suggested that the proapoptotic impacts of quercetin, which led to the downregulation of cellular MCL-1 and survivin levels, might commonly occur in B lymphoma and T-ALL Jurkat cells.

Consequently, these results indicated that quercetin-induced activation of the intrinsic mitochondrial apoptosis pathway was exerted by DNA damage-mediated activation of the ATM/ATR-CHK1/CHK2-p53 pathway and resultant increase of NOXA level and decrease of BAG3, MCL-1, and survivin levels, which predisposed cells to undergo BCL-XL-sensitive BAK activation, *Δψ*m loss, and subsequent mitochondria damage-mediated caspase cascade activation.

### 3.3. Involvement of ROS Generation in Quercetin-Induced Apoptotic Responses

**Figure 4:**
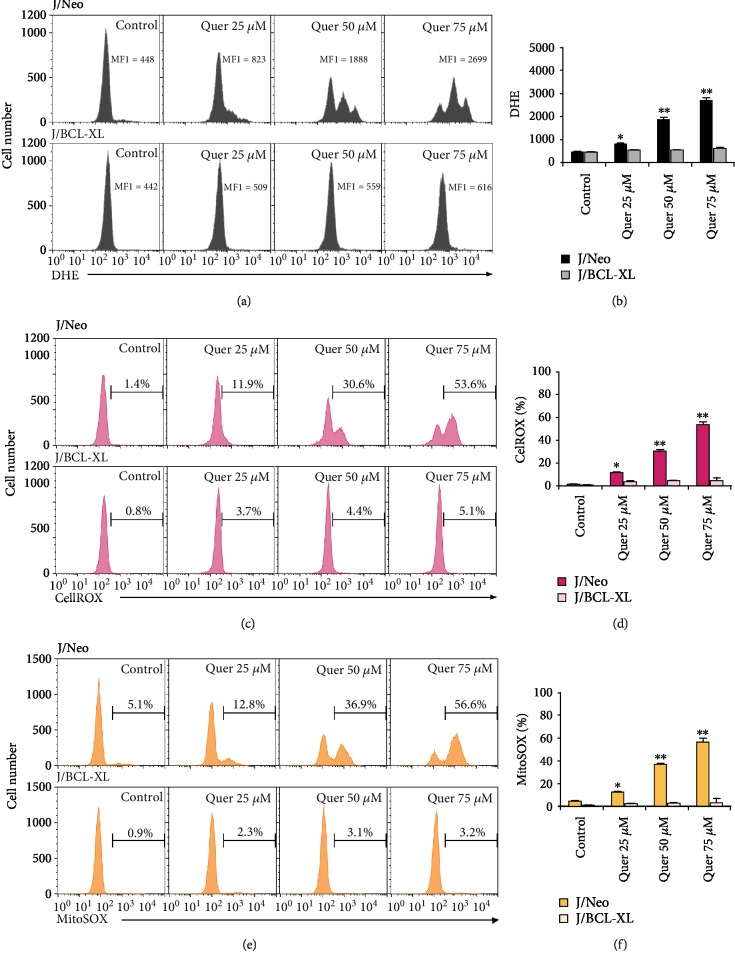
Cytosolic and mitochondrial ROS levels were commonly enhanced in quercetin-treated J/Neo cells but not in J/BCL-XL cells. (a, b) The intracellular ROS, (c, d) cytosolic ROS, and (e, f) mitochondrial ROS levels in J/Neo and J/BCL-XL cells treated with vehicle or quercetin at indicated doses for 11 h were analyzed using flow cytometry with DHE, CellROX Deep Red, and MitoSOX Red staining, respectively, and indicated by the MFI or percentage of the cells. A representative study is shown and two additional experiments yielded similar results. Error bars represent standard deviations with ^∗^ and ^∗∗^ indicating *P* < 0.05 and *P* < 0.01, respectively, compared with the control.

Although quercetin is a well-known flavonoid antioxidant and has been reported to exert cytoprotective roles in oxidative stress-mediated apoptotic conditions [48, 49], it has also been shown to cause intracellular ROS generation, which leads to apoptotic cell death in human hepatoma cells [50], human promyelocytic leukemia HL-60 cells [51], and human bladder cancer cells [29]. To examine whether ROS generation is associated with quercetin-induced intrinsic mitochondrial apoptosis in Jurkat T cells, changes in intracellular ROS levels between quercetin-treated J/Neo and J/BCL-XL cells were compared by flow cytometry using DHE staining. As shown in Figures 4(a) and 4(b), the mean fluorescent intensity (MFI) value of vehicle-treated J/Neo cells (the control) was 448, whereas that of quercetin-treated J/Neo cells at concentrations of 25, 50, and 75 *μ*M was 823, 1888, and 2699, respectively. Under these conditions, quercetin failed to increase the MFI values in J/BCL-XL cells, demonstrating that the ROS generation in quercetin-treated Jurkat T cells was targeted by antiapoptotic action of BCL-XL.

The quercetin-caused ROS generation in J/Neo and J/BCL-XL cells was further analyzed using the CellROX Red cytosolic ROS indicator [52] and the MitoSOX Red mitochondrial superoxide indicator [53]. Flow cytometric data revealed that quercetin treatment in J/Neo cells resulted in a significant elevation in both cytosolic and mitochondrial ROS levels in a dose-dependent manner; however, the quercetin-caused increases in cytosolic and mitochondrial ROS levels were barely or not detected in J/BCL-XL cells (Figures 4(c)–4(f)). As the antiapoptotic action of BCL-XL centers in its blocking of mitochondrial damage to release mitochondrial cytochrome *c* into the cytosol and thus preventing of cytochrome *c*-mediated caspase cascade activation [54, 55], these previous and current results suggested that quercetin-caused intracellular ROS generation might be a downstream event of mitochondrial damage and dysfunction, which can be prevented by overexpressed BCL-XL, rather than a proximal event provoking DNA damage- and/or mitochondrial damage-dependent apoptosis.

### 3.4. Quercetin-Induced Apoptotic Responses in Wild-Type (Clone A3), FADD-Deficient (Clone I2.1), and Caspase-8-Deficient (Clone I9.2) Jurkat T Cells

The activation of caspase-8 plays an essential role as the initiator caspase in the extrinsic death receptor- (DR-) mediated apoptosis signaling pathway [56]. In addition, caspase-8 activation is known to occur as a downstream event of the intrinsic mitochondrial/caspase-9/caspase-3 apoptosis pathway to comprise a positive feedback loop involving tBID-mediated mitochondrial cytochrome *c* release in drug-induced apoptosis of tumor cells [57, 58]. To examine whether the death receptor (DR)/DR ligand system is involved in quercetin-induced apoptosis in Jurkat T cells, we compared the apoptotic effect of quercetin on A3 cells with those on I2.1 and I9.2 cells, both of which are known to be refractory to the extrinsic DR-dependent apoptosis [59].

**Figure 5:**
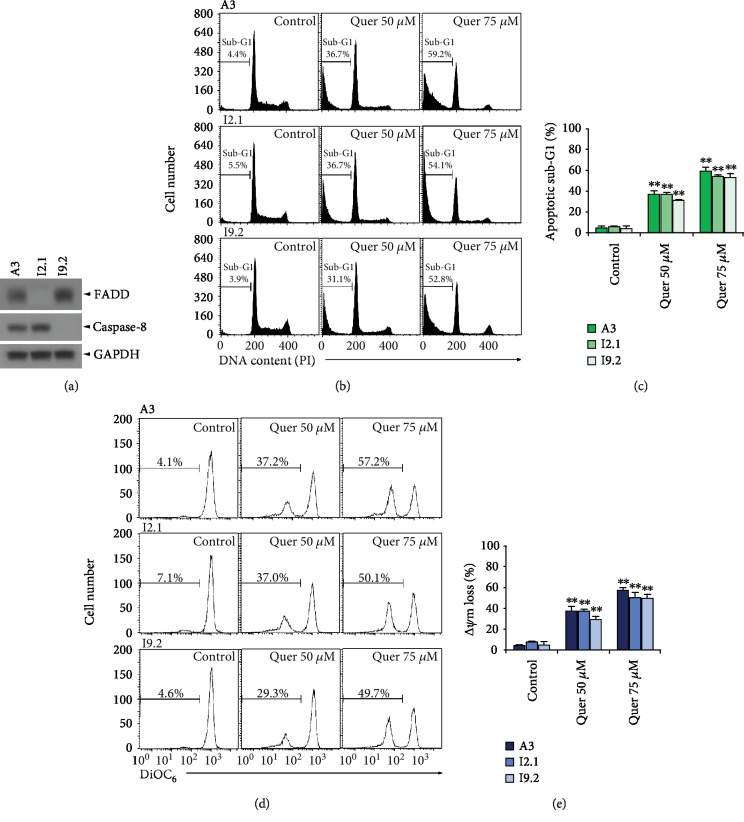
The sensitivity of FADD- and capase-8-positive wild-type Jurkat clone A3 to the apoptogenic activity of quercetin was similar to that of FADD-deficient Jurkat T cell clone I2.1 and caspase-8-deficient Jurkat T cell clone I9.2. (a) Exponentially growing individual cells were subjected to western blot analyses of caspase-8, FADD, and GAPDH as described in Materials and Methods. (b–e) After A3, I2.1, or I9.2 cells (5 × 10^5^ cells/ml) were incubated with vehicle or quercetin at indicated doses for 11 h, the cells were stained with PI and with DiOC_6_ for flow cytometric analysis of the cell cycle state and *Δψ*m loss, respectively, as described in Materials and Methods. A representative study is shown and two additional experiments yielded similar results. Error bars represent standard deviations with ^∗∗^ indicating *P* < 0.01, compared with the control.

As shown in Figure 5(a), results of western blot analysis showed that although Jurkat A3 appeared to express both FADD and caspase-8, I2.1 and I9.2 cells failed to express FADD and caspase-8, respectively. Following treatment with 50 and 75 *μ*M quercetin for 12 h, apoptotic sub-G_1_ cells were accumulated to a similar extent in A3, I2.1, and I9.2 cells (Figures 5(b) and 5(c)). Simultaneously, the *Δψ*m loss induced in A3, I2.1, and I2.1 cells after quercetin treatment was similar to each other (Figures 5(d) and 5(e)). These results confirmed that quercetin-induced apoptosis was provoked by the intrinsic mitochondrial apoptosis pathway, which proceeded independently of the extrinsic apoptosis pathway.

### 3.5. Concomitant Induction of Cytoprotective Autophagy and Apoptosis by Quercetin

In cells under normal conditions, autophagic events are generally suppressed. However, the autophagic response occurs under stress conditions including energy starvation or antitumor drug treatment [60]. As compared to normal counterpart, tumor cells frequently possess an elevated autophagic capacity, allowing tumor cells to be resistant to chemotherapy [60, 61]. Recently, quercetin has been reported to induce apoptosis and protective autophagy in human cancer cells, such as gastric cancer cells [62], glioblastoma U373MG cells [28], cervical cancer HeLa cells [63], and bladder cancer cells [29]. To further understand the antitumor cytotoxicity of quercetin, the molecular signaling pathways linking quercetin-induced apoptosis and autophagy in J/Neo and J/BCL-XL cells were investigated. Because the acidic vesicular organelles (AVOs) or autolysosome vacuoles are known to be formed by autophagosome-lysosome fusion as a key feature of autophagy [64], flow cytometric analyses were carried out to detect if the formation of AVOs or autolysosome vacuoles is elevated in quercetin-treated J/Neo and J/BCL-XL cells.

**Figure 6:**
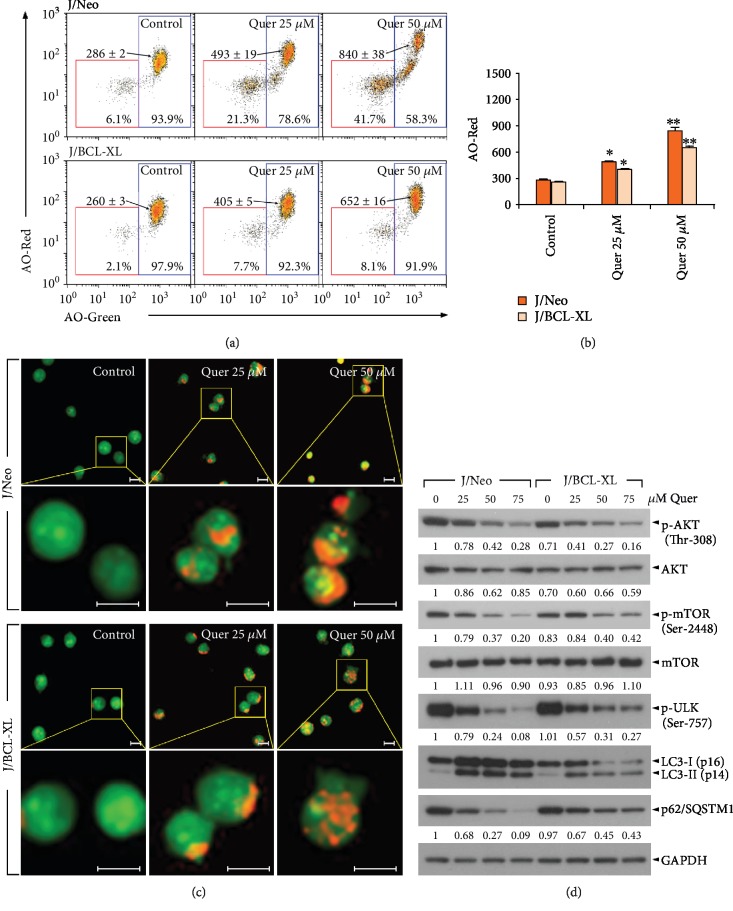
Quercetin induces autophagy in J/Neo and J/BCL-XL cells. (a–c) After cells were treated with vehicle or quercetin at indicated doses (25 and 50 *μ*M) for 11 h, the enhancement of acridine orange- (AO-) Red fluorescence was analyzed by flow cytometry and fluorescence microscopy, respectively. The AO-Red fluorescence indicates the formation of AVOs and autolysosome vacuoles, resulting from autophagy induction, and the AO-Green fluorescence indicates AO staining of DNA/RNA in cells. Error bars represent standard deviations with ^∗^ and ^∗∗^ indicating *P* < 0.05 and *P* < 0.01, respectively, compared with the control. (d) Western blot analyses of p-AKT (Thr-308), AKT, p-mTOR (Ser-2448), mTOR, p-ULK (Ser-757), LC3-I/LC3-II, p62/SQSTM1, and GAPDH were performed as described in Materials and Methods. A representative study is shown and two additional experiments yielded similar results.

As shown in Figures 6(a) and 6(b), while acridine orange could effectively stain the cellular DNA/RNA in green (AO-Green) and the AVOs or autolysosome vacuoles in red (AO-Red), a significant dose-dependent enhancement of the AO red fluorescence was observed in quercetin-treated (25 and 50 *μ*M) J/Neo and J/BCL-XL cells, suggesting that the AVOs or autolysosome vacuoles were formed by quercetin irrespective of the presence of overexpressed BCL-XL. At the same time, treatment of J/Neo cells with 25 and 50 *μ*M quercetin resulted in the accumulation of low AO-Green/low AO-Red cells to the levels of 21.3% and 41.7%, respectively; however, the increase of low AO-Green/low AO-Red cells was not or barely detected in J/BCL-XL cells. The percentages of these subpopulations, which are characterized by low AO-Green/low AO-red fluorescence, were similar to the percentages of apoptotic sub-G_1_ cells detected by flow cytometric analysis with PI staining (data not shown). This suggested that the low AO-Green/low AO-Red cells might be late apoptotic cells possessing apoptotic DNA fragmentation and impairments in AO-stainable acidic compartments.

Fluorescence microscopy also exhibited a remarkable elevation in quercetin-induced formation of AVOs in J/Neo and J/BCL-XL cells (Figure 6(c)). Under these conditions, western blot analysis revealed that the autophagy-associated alteration of two autophagy marker proteins, such as conversion of microtubule-associated protein 1 light chain 3-I (LC3-I, 16 kDa) into LC3-II (14 kDa) and downregulation of the polyubiquitin-binding protein p62/sequestosome 1 (p62/SQSTM1) level, was induced in quercetin-treated J/Neo and J/BCL-XL cells (Figure 6(d)). Furthermore, AKT-mTOR pathway inhibition, as evidenced by reduction in the levels of p-AKT (Thr-308), p-mTOR (Ser-2448), and p-ULK (Ser-757), was observed to a similar level in both cell types following quercetin treatment. These results indicated that quercetin caused autophagy via suppression of the AKT-mTOR pathway in Jurkat T cells, regardless of the overexpression of BCL-XL. It is noteworthy that because the AKT pathway is also known to play an important role in cell survival by mediating inhibitory phosphorylation of proapoptotic protein BAD, it cannot exclude a possible contribution of the AKT inhibition, causing promotion of apoptosis, in quercetin-induced cell death [26, 65, 66].

**Figure 7:**
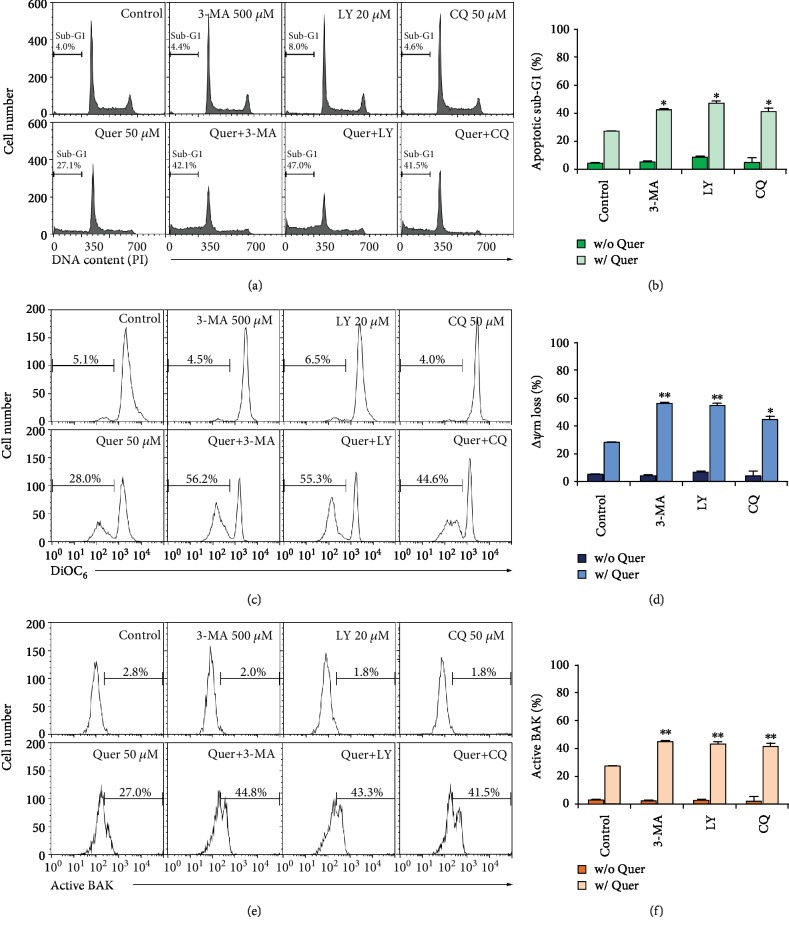
Cotreatment of J/Neo cells with pharmacological inhibitors (3-MA, LY, or CQ) of autophagy promotes quercetin-induced sub-G_1_ cell accumulation, *Δψ*m loss, and BAK activation. (a–f) After treatment with 50 *μ*M quercetin in the absence or presence of either 500 *μ*M 3-MA, 20 *μ*M LY, or 50 *μ*M CQ for 7 h, the cells were harvested and subjected to flow cytometry to analyze the percentage of apoptotic sub-G_1_ cells, *Δψ*m loss, and BAK activation. A representative study is shown and two additional experiments yielded similar results. Error bars represent standard deviations with ^∗^ and ^∗∗^ indicating *P* < 0.05 and *P* < 0.01, respectively, compared with the control.

**Figure 8:**
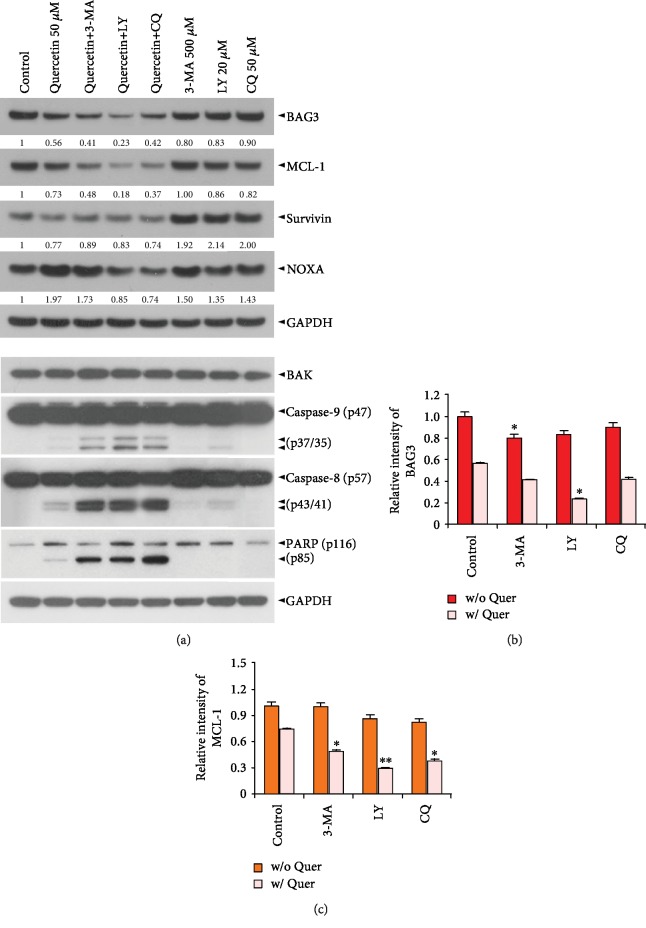
Cotreatment of J/Neo cells with pharmacological inhibitors (3-MA, LY, or CQ) of autophagy promotes quercetin-induced downregulation of BAG3 and MCL-1 levels, caspase-9/caspase-8 activation, and PARP cleavage. (a) After treatment with 50 *μ*M quercetin in the absence or presence of either 500 *μ*M 3-MA, 20 *μ*M LY, or 50 *μ*M CQ for 7 h, the cells were harvested and subjected to western blot analyses of BAG3, MCL-1, survivin, NOXA, BAK, caspase-9, caspase-8, PARP, and GAPDH as described in Materials and Methods. A representative study is shown and two additional experiments yielded similar results. (b, c) The arbitrary densitometric units of BAG3 and MCL-1 were normalized to those of GAPDH. Error bars represent standard deviations with ^∗^ and ^∗∗^ indicating *P* < 0.05 and *P* < 0.01, respectively, compared with the control.

To examine the relationship between quercetin-induced autophagy and apoptosis, the effect of the pharmacologic inhibitors of autophagy on quercetin-induced sub-G_1_ cell accumulation and *Δψ*m loss was investigated in J/Neo cells. The autophagy inhibitors 3-methyladenine (3-MA) and LY294002 (LY) can inhibit the class III PI3K activity required for the formation of autophagosomes during autophagy process [67], whereas the autophagy inhibitor chloroquine (CQ) blocks autolysosome formation in the late stage of autophagy [68]. After treatment of J/Neo cells with 50 *μ*M quercetin for 7 h, the induced apoptotic sub-G_1_ peak and *Δψ*m loss were 27.1% and 28.0%, respectively (Figures 7(a)−7(d)). The quercetin-induced apoptotic sub-G_1_ peak was enhanced to the levels of 42.1%, 47.0%, and 41.5% in the presence of 500 *μ*M 3-MA, 20 *μ*M LY, and 50 *μ*M CQ, respectively. The quercetin-induced *Δψ*m loss was also elevated to the levels of 56.2%, 55.3%, and 44.6% by 500 *μ*M 3-MA, 20 *μ*M LY, and 50 *μ*M CQ, respectively. Additionally, quercetin-induced BAK activation was markedly enhanced by 3-MA, LY, or CQ (Figures 7(e) and 7(f)). Furthermore, results of western blot analyses revealed that quercetin-induced apoptotic events, including reduction of BAG3 and MCL-1 levels, caspase-9/caspase-8 activation, and PARP cleavage, were markedly elevated in the presence of 3-MA, LY294002, or CQ in common (Figures 8(a)–8(c)). However, the level of BAK protein remained constant.

Consequently, these results demonstrate that the autophagic events provoked in J/Neo cells being treated with quercetin were a cellular response to sustain cell survival from mitochondrial damage-mediated apoptotic cell death via reducing the quercetin-induced BAK activation.

## 4. Conclusion

This study demonstrated that the cytotoxic effect of quercetin (25–75 *μ*M) on Jurkat T cells was exerted by inducing intrinsic mitochondrial apoptosis via sequentially mediating the activation of the ATM-CHK1/CHK2-p53 pathway, upregulation of proapoptotic NOXA level, downregulation of antiapoptotic BAG3, MCL-1, and survivin levels, BAK activation, *Δψ*m loss, ROS generation, caspase cascade activation, PARP cleavage, and apoptotic DNA fragmentation. These apoptogenic actions of quercetin, except for upstream events of the mitochondrial damage-dependent apoptosis pathway, are completely blocked by the overexpression of BCL-XL. Quercetin induces autophagy resulting from attenuating the AKT-mTOR pathway, leading to the manifestation of several cellular autophagy markers including increased formation of acridine orange-stainable AVOs, LC3-I/LC3-II conversion, and p62/SQSTM1 downregulation. However, none of these quercetin-caused autophagic events are abrogated by BCL-XL overexpression. Cotreatment with a pharmacological autophagy inhibitor (3-MA, LY, or CQ) enhances the quercetin-induced reduction of BAG3 and MCL-1 levels, BAK activation, and subsequent promotion of mitochondrial damage-dependent apoptosis. These results suggested that combining quercetin with an autophagy inhibitor may be a promising antitumor treatment of human T-ALL.

## Data Availability

The data used to support the findings of this study are included within the article.

## Supplementary Materials

Supplementary Materials Supplementary Table 1: dry weights of the 95% ethanol extract and its organic solvent fractions prepared from the grains of Sorghum bicolor (L.) Moench var. Hwanggeumchal. Supplementary Table 2: HPLC-dependent analysis of phenolic components in 95% EtOH extract and its organic solvent fractions from the grains of Sorghum bicolor (L.) Moench var. Hwanggeumchal. Supplementary Table 3: inhibitory effect of the major phenolic compounds in organic solvent fractions prepared from the grains of Sorghum bicolor (L.) Moench var. Hwanggeumchal on proliferation of human T-ALL Jurkat cells. Graphic abstract.
