# Supplementary material for: Enhancement of Quercetin-Induced Apoptosis by Cotreatment with Autophagy Inhibitor Is Associated with Augmentation of BAK-Dependent Mitochondrial Pathway in Jurkat T Cells
Source: Oxid Med Cell Longev. 2019 Nov 15;2019:7989276. doi: 10.1155/2019/7989276 (PMC6885204; doi:10.1155/2019/7989276)
Supplement: Supplementary Materials — Supplementary Table 1: dry weights of the 95% ethanol extract and its organic solvent fractions prepared from the grains of Sorghum bicolor (L.) Moench var. Hwanggeumchal. Supplementary Table 2: HPLC-dependent analysis of phenolic components in 95% EtOH extract and its organic solvent fractions from the grains of Sorghum bicolor (L.) Moench var. Hwanggeumchal. Supplementary Table 3: inhibitory effect of the major phenolic compounds in organic solvent fractions prepared from the grains of Sorghum bicolor (L.) Moench var. Hwanggeumchal on proliferation of human T-ALL Jurkat cells. Graphic abstract. [file 7989276.f1.docx]

**Supplementary Table 1.** Dry weights of the 95% ethanol extract and its organic solvent fractions prepared from the grains of *Sorghum bicolor* (L.) Moench var. *Hwanggeumchal*.

| *S. bicolor* var. *Hwanggeumchal grains*  (dry weight, kg) | Recovered amounts (dry weight, g) | | | | |
| --- | --- | --- | --- | --- | --- |
|  | 95% EtOH*  extract | Hexane  fraction | MC*  fraction | EtOAc* fraction | BuOH*  fraction |
| 30.0 | 850.0 | 444.1 | 59.3 | 47.2 | 169.5 |

*Symbols: EtOH, ethanol; MC, methylene chloride; EtOAc, ethyl acetate; BuOH, butanol.

**Supplementary Table 2.** HPLC-dependent analysis of phenolic components in 95% EtOH extract and its organic solvent fractions from the grains of *Sorghum bicolor* (L.) Moench var. *Hwanggeumchal*.

| No. | Phenolic compounds | Contents (μg/mg of extract) | | | | |
| --- | --- | --- | --- | --- | --- | --- |
|  |  | 95% EtOH*  extract | Hexane  fraction | MC*  fraction | EtOAc*  fraction | BuOH*  fraction |
| 1 | Biochanin A | 0.21 | 0.15 | 0.14 | N.D** | N.D |
| 2 | Caffeic acid | 0.49 | 0.05 | 0.08 | 1.42 | N.D |
| 3 | *t*-Cinnamic acid | 0.45 | 0.08 | 0.09 | 0.14 | 0.06 |
| 4 | *p*-Coumaric acid | 0.25 | 0.07 | 0.06 | 0.68 | 0.82 |
| 5 | Gentisic acid | 2.13 | N.D | 8.73 | N.D | 1.16 |
| 6 | Hesperidin | 1.10 | 0.46 | 0.42 | 3.91 | N.D |
| 7 | Hesperitin | 0.33 | 0.22 | 2.90 | 0.31 | 0.80 |
| 8 | 4-Hydroxybenzoic acid | 0.32 | 0.06 | 0.06 | 2.62 | N.D |
| 9 | Kaempferol | 2.90 | 0.43 | 2.53 | 1.17 | 6.88 |
| 10 | Myricetin | 0.23 | N.D | N.D | 7.00 | 0.38 |
| 11 | Naringenin | 2.60 | 0.49 | 4.50 | 1.59 | 2.09 |
| 12 | Naringin | 1.33 | N.D | N.D | 1.84 | 1.33 |
| 13 | Protocatechuic acid | 1.31 | 1.00 | 0.94 | N.D | N.D |
| 14 | Quercetin | 9.29 | 4.66 | 1.11 | 7.27 | 2.13 |
| 15 | Resveratrol | 1.40 | 0.51 | 0.69 | 0.44 | N.D |
| 16 | Salicylic acid | 1.76 | 1.90 | 1.83 | 1.73 | 0.21 |
| 17 | Veratric acid | 1.10 | 0.36 | 0.33 | 4.83 | 0.22 |

*Symbols: EtOH, ethanol; MC, methylene chloride; EtOAc, ethyl acetate; BuOH, butanol

**N.D; not detected

**Supplementary Table 3.** Inhibitory effect of the major phenolic compounds in organic solvent fractions prepared from the grains of *Sorghum bicolor* (L.) Moench var. *Hwanggeumchal* on proliferation of human T-ALL Jurkat cells.

| Cell lines | IC_50_ (μg/ml)* | | | | | |
| --- | --- | --- | --- | --- | --- | --- |
|  | Quercetin | Gentisic acid | Naringenin | Kaempferol | Resveratrol | Salicyclic acid |
| Jurkat  J/Neo cells | 33.3 ± 0.8 | >200.0 | >200.0 | 112.6 ± 2.6 | 121.5 ± 3.3 | >200 |
| Jurkat  A3 cells | 36.0 ± 1.9 | >200.0 | >200.0 | 96.6 ± 4.9 | 107.4 ± 4.3 | >200 |

*The IC_50_ value indicates a concentration of each compound, which caused 50% reduction in cell viability based on MTT assay. The cells (J/Neo, 5 × 10^4^/well; A3, 7.5 × 10^4^ cells/well) were cultured with different concentrations of compounds for 48 h and the final 4 h was incubated with MTT solution to assess cell viability.

**Graphic abstract**

**
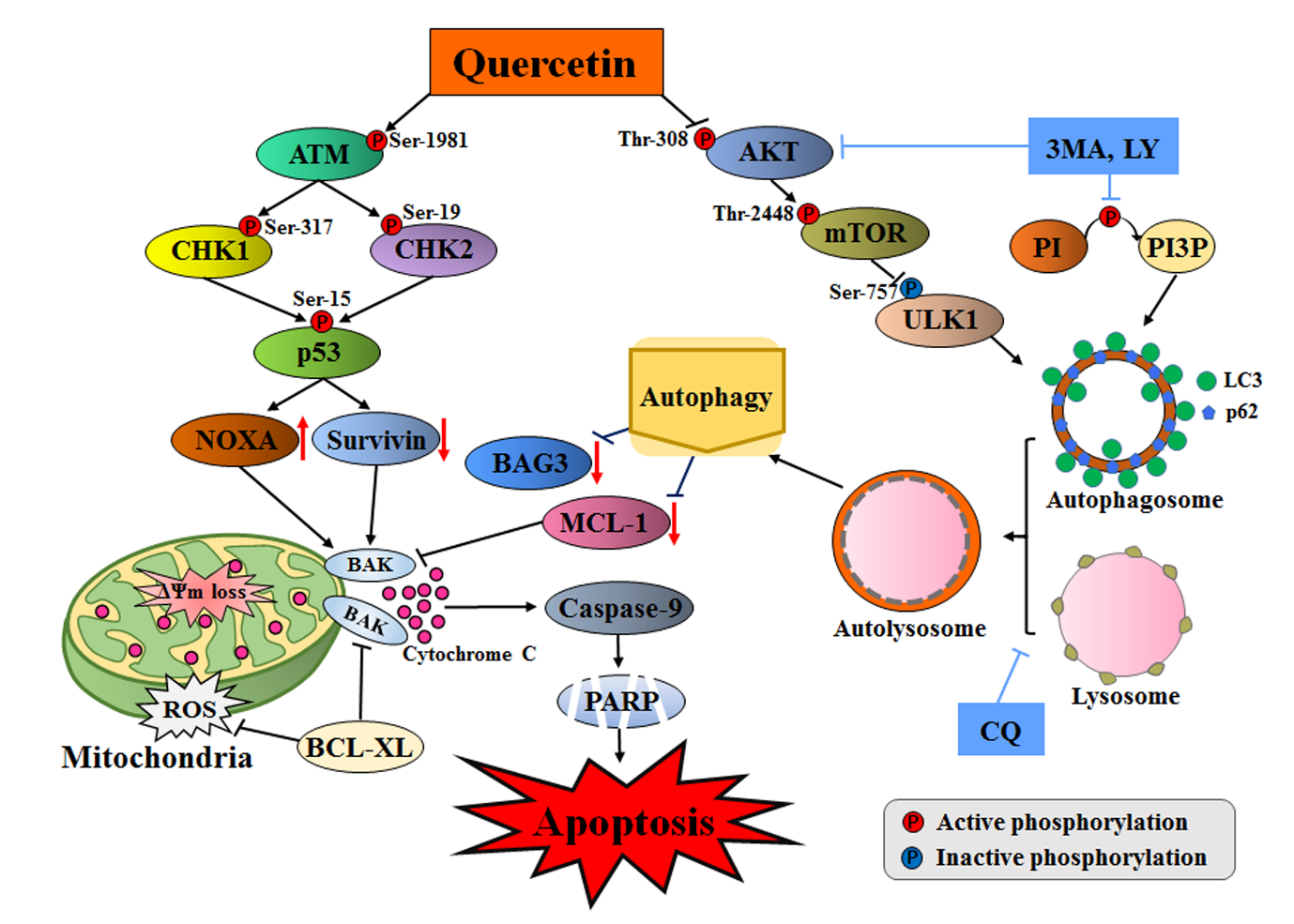
**
